# Promoter Hypermethylation of Tumor Suppressor Genes Located on Short Arm of the Chromosome 3 as Potential Biomarker for the Diagnosis of Nasopharyngeal Carcinoma

**DOI:** 10.3390/diagnostics11081404

**Published:** 2021-08-03

**Authors:** Thuan Duc Lao, Toan Ngoc Nguyen, Thuy Ai Huyen Le

**Affiliations:** Department of Pharmaceutical and Medical Biotechnology, Faculty of Biotechnology, Ho Chi Minh City Open University, Ho Chi Minh City 700000, Vietnam; thuan.ld@ou.edu.vn (T.D.L.); nngoctoan2000@gmail.com (T.N.N.)

**Keywords:** nasopharyngeal carcinoma, chromosome 3p, methylation, diagnosis

## Abstract

DNA methylation, the most common epigenetic alteration, has been proven to play important roles in nasopharyngeal carcinoma (NPC). Numerous tumor suppressor genes located on the chromosome 3p, particularly in the region of 3p21, are frequently methylated in NPC, thus suggesting great potential for diagnosis of NPC. In this review, we summarize recent findings of tumor suppressor genes on chromosome 3 that likely drive nasopharyngeal tumor development and progression, based on previous studies related to the hypermethylation of these target genes. Better understanding will allow us to design further experiments to establish a potential test for diagnosis of NPC, as well as bring about methylated therapies to improve the treatment of NPC.

## 1. Introduction

Cancer is a serious global health problem and the second leading cause of death globally, with an estimated 9.6 million deaths in 2018. It was noted that the number of new cases will reach 23.6 million by 2030 [1]. Nasopharyngeal carcinoma (NPC) is the most common cancer in the area of head and neck cancer. NPC has unbalanced geographical differences in distribution, which gravitates toward Asia. Asian countries account for 84.62% of the global burden of NPC. Similarly, the highest mortality rate has also been observed in Asian countries, accounting for 85.36% of the global mortality of NPC. In recent decades, even though improvements in nasopharyngeal cancer treatment have made great progress, diagnosis at the advanced stage of tumors often leads to treatment failure as well as reduction in the survival of patients. Early diagnosis represents a beneficial opportunity to improve the treatment results, resulting in a high cure rate [2]. The paucity or non-specific symptoms of the early stage of NPC, as well as the deeply seated location of the nasopharynx, are major obstacles leading to the diagnosis of NPC at the advanced stage. Finding the effective biomarkers for early diagnosis of NPC will contribute significantly to improving survival and will guide the choice of subsequent therapy, particularly in the countries with high incidence.

Providing information about the etiological factors in the prevention program is very important. At present, much effort has been made to identify early biomarkers by focusing on the etiological factors that lead to nasopharyngeal tumorigenesis. NPC presents as a multiple etiological factors disease that is caused by: (1) the infection of Epstein-Barr virus (EBV); (2) environmental factors; and (3) genetic susceptibility [3]. Recent advances in the field of molecular and cytogenetic studies have shown that genetic and epigenetic events play key roles at all stages of tumorigenesis and promote cancer progression [4]. It has been suggested that the mechanism of epigenetic alteration may act as the initiating event for various human cancers, including NPC, through regulating cell signal pathways [4,5,6,7]. Epigenetics, originally defined by C.H. Waddington (1942), refers to “the causal interactions between genes and their products, which bring the phenotype into being”, and involves the following main categories: DNA methylation, covalent histone modifications, and non-coding RNAs [4,8]. Unlike genetic mutation, epigenetic alterations can be reversed to their normal state, thereby making such initiatives promising therapeutic biomarkers [4]. DNA methylation, which is the most common epigenetic alteration, is a covalent modification of 5′ cytosine located at a CpG dinucleotide by adding a methyl group in the 5th carbon of the ring using S-adenosyl methionine as a methyl donor [4,9,10]. The alterations of DNA hypermethylation in the promoter region or the first exon of the tumor suppressor gene (TSG) lead to its inactivation. Decreasing of TSG is proven to be significantly associated with tumorigenesis. Therefore, understanding the characteristics of DNA hypermethylation could expand prospects for the development of early diagnostic biomarkers.

## 2. The Inactivation of Tumor Suppressor Genes on Chromosome 3

The current genomic changes in NPC detected by genome-wide approaches such as allelotype studies and comparative genomic hybridization have identified high frequencies of genetic imbalances on various chromosomes such as 1q, 2q, 3p, 9p, 11p, 12q, 13q, 14q, 16q, and 17q [11,12,13,14]. Among the chromosome abnormalities, the silencing of TSGs on chromosome 3 is a frequent event in nasopharyngeal tumorigenesis [13,15,16,17]. These findings suggest that inactivation of TSGs on chromosome 3 is an early and crucial event in NPC development and progress [13]. In this review, we summarize the function of TSG on chromosome 3 to understand the role of TSGs on chromosome 3 in the development of NPC, suggested to be a promising target for early diagnostic biomarkers. The most important epigenetic event is the hypermethylation at multiple chromosome 3p TSG. The silencing of TSGs on chromosome 3p, including *ADAM*, *BLU*, *DLEC1*, *GNAT1, LARS2*, *LTF*, *MLH1, RASSF1A*, *RAR-β*, *TIG1*, *VLH1*, *PTPRG*, and *ITGA9*, by the phenomenon of DNA aberrant hypermethylation in NPC, seems to be the key role in the mechanism of NPC tumorigenesis, as reported. Roles of TSGs on chromosome 3 during nasopharyngeal carcinogenesis, including cell proliferation, cell cycle regulation, apoptosis, etc., have been reported in recent years (Table 1).

Among regions belonging to 3p, multiple genes located at the region of 3p21.3 have been widely studied in NPC xenografts and cell lines and in primary tumors. Based on previous studies, shown in Table 1, *RASSF1A* was reported to be the most studied TSG involved in the tumorigenesis of nasopharynx.

## 3. *RASSF1A* and Panel of Genes as Methylation Biomarkers for NPC

**Figure 1:**
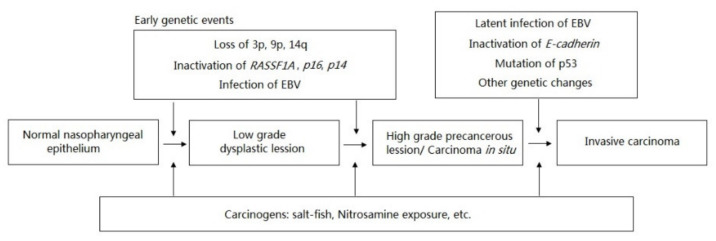
Inactivation of *RASSF1A*—the early genetic event driving NPC.

*Ras Association domain Family 1A* (*RASSF1A*) gene, located on chromosome 3p21.3, was first mapped by Dammann et al. (2000). They reported that the inactivated expression of RASSF1A transcript was frequently observed in lung cancer by the mechanism of hypermethylation [42]. The inactivation of *RASSF1A* has been identified as one of the early events that drives to nasopharyngeal carcinoma (Figure 1) [3,43]. It was reported that RASSF1A induces cell-cycle arrest in the phase of G0/G1 by inhibiting the accumulation of cyclin D1; thus, *RASSF1A* blocks the cell cycle progression. RASSF1A is suggested to be an important human tumor suppressor protein acting at the level of G1/S-phase cell cycle progression [44]. Later on, a significant correlation between the methylation status of *RASSF1A* with NPC was reported in the NPC cell lines, xenografts, and primary NPC tumors collected from Asian patients, but not in the normal nasopharyngeal epithelia [34]. In their report, they investigated the finding that the 5′ CpG of *RASSF1A*’s promoter hypermethylation was detected in 75% of cell lines (C666-1, CNE-1, and CNE-2), 100% of xenografts (xeno-2117, xeno-1915, xeno-8, and xeno-666), and 66.67% of primary tumors. Specifically, no *RASSF1A* expression was detected on cell lines as well as xenografts. The re-expression of *RASSF1A* in cell lines was detected after treatment with 5’-aza-2’deoxycytidine [34]. In the study of Wang et al. (2009), the expression of *RASSF1A* was downregulated in the cell line of CNE-2 due to promoter hypermethylation. The loss of *RASSF1A* expression was greatly restored by the treatment of 5’-aza-2’deoxycytidine [33]. Again, according to the systematic reviews and meta-analyses by Ye et al. (2017), they concluded that methylation of *RASSF1A* promoter may be associated with the development, progression, and metastasis of NPC through the evaluation of the pooled sensitivity, specificity, and AUC of *RASSF1A* promoter methylation in NPC samples vs. non-tumor samples [45]. Additionally, hypermethylation of *RASSF1A* was reported as a frequent and significant event in EBV-positive NPC, and may be an important event in the pathogenesis of EBV-infected NPC. Moreover, the hypermethylation of *RASSF1A* could serve as a potential biomarker for worse overall survival [46]. Based on these results, promoter hypermethylation of *RASSF1A*, leading to transcriptional inactivation, acts as the critical target gene involved in nasopharyngeal tumorigenesis. Further functional evidence supporting *RASSF1A* as the potential candidate for the NPC targeting strategy was demonstrated in the in vivo BALB/c nude mice assay. In their report, tumors were dramatically reduced in the *RASSF1A*-tranfected nude mice [13]. Taken together, these findings suggest that *RASSF1A* is the best candidate tumor suppressor at 3p21.3 in NPC.

The specificity, sensitivity, positive predictive value, and negative predictive value of methylated *RASSF1A*-based diagnosis vary from 87.14% to 100.00%, 0.00% to 91.18%, 0.00% to 100.00%, and 25.00% to 72.62%, respectively (Table 2). The low sensitivity and specificity of a single-gene biomarker limits the use of one gene for cancer diagnosis. Therefore, for the aim of increasing the diagnosis power in NPC, DNA methylation of a panel of different genes combined with *RASSF1A* was analyzed. Given the evidence of methylation recorded in NPC, a panel of other useful methylated biomarkers for NPC is summarized in Table 3. Taking into account that at least one gene was methylated, gains in specificity, sensitivity, positive predictive value, and negative predictive value of diagnosis, compared to corresponding single-gene *RASSF1A*, were recorded. When the methylation of at least one gene was considered, all values could increase to a value of 100%. Kwong et al. (2002) examined the methylation of eight genes, including *RASSF1A*, *RAR-β*, *DAPK*, *p16*, *p15*, *p14*, *MGMT*, and *GSTP1*, in primary tumors and normal epithelium. They reported that no methylation of these genes was detected in the six normal nasopharyngeal epithelium samples. In the case of primary tumors, only one gene of *GSTP1* was unmethylated. They found that aberrant methylation in at least one of the seven genes, including *RASSF1A*, *RAR-β*, *DAPK*, *p16*, *p15*, *p14*, and *MGMT,* was detectable in 100% of the tumors of patients; specificity, sensitivity, positive predictive value, and negative predictive value reached 100% for each value [30].

According to the World Health Organization, pathological evidence found by biopsies is the gold standard for NPC diagnosis [47]. Biopsy-based diagnosis faces difficulty in that the symptoms of NPC are not conclusive at early stages. Additionally, almost patients were diagnosed at the advanced stage. Therefore, searching the minimally invasive biomarkers for the early stage, as well as monitoring the disease progression, is an urgent need. A variety of non-invasive or minimally invasive sampling methods have been attempted for the development of the non-invasive diagnosis of NPC. A panel of methylation markers consisting of *RASSF1A* and other TSGs has been proposed as the complementary test for diagnosis of NPC based on the minimal invasive or non-invasive samples, including plasma, M&T rinsing fluid, buffy coat, and nasopharyngeal brushing [5,26,29,31,32]. For example, in the study of Chang et al. (2003), they compared the power of methylation markers on different sources of samples; as a result, the panel of five methylation markers including *RASSF1A*, *E-cadherin*, *DAPK*, *p15*, and *p16* had a specificity of 100.00% and sensitivity of 80.00% [29]. Hutajulu et al. (2011) reported that a panel of methylation biomarkers consisting of *RASSF1A*, *CHFR*, *p16*, *RIZ1*, and *WIF1* showed high frequency of at least one methylated gene of NPC compared to individual markers, providing good discrimination between NPC and non-cancer [5].

Circulating cell-free Epstein-Barr virus (EBV) levels have emerged as a promising biomarker in clinical decision making and improving NPC treatment [28,31,37,48]. It could be explained that EBV DNA load has been implied as the factor that contributes the methylation alterations through the activation of DNA methyltransferase by EBV LMP-1, which involves c-JUN NH (2)-terminal kinase signaling or interaction with transcriptional repression [49,50]. Hence, analysis of methylation status of selected TSGs is proposed as the additive test to EBV-DNA detected assay for early diagnosis of NPC [5,51]. Zhou et al. (2005) reported that higher frequency of *RASSF1A* methylation and higher viral load were detected in the T tissue (containing more than 70% of tumor cells) compared to P and Z tissues (located 0.5 and 1.0 cm outside of visible NPC lesions, respectively). They concluded that hypermethylation of *RASSF1A* and high EBV load might be important events in NPC pathogenesis, and they may be useful molecular diagnostic markers for NPC [37]. The method of multiplex methylation-specific PCR, which could simultaneously detect methylation of *RASSF1A* and *DAPK* and the presence of EBV DNA (*EBNA-1* and *LMP1*), using only picograms of tumor DNA from NP swabs, has been developed. The specificity and sensitivity of this method are 100% and 98%, respectively, in detecting 49 NPCs which include 19 early-stage patients [32]. Therefore, these studies opened the way to develop innovative diagnosis strategies for NPC.

The last decades have seen the development of a plethora of drugs that were designed to specifically target the phenomenon of DNA aberrant hypermethylation, which was identified as the driver of cancer development, including NPC. To date, two nucleoside analogues, including 5-azacytidine and 5’-aza-2’deoxycytidine, have been approved as therapeutic agents for cancers [52]. Mechanically, these drugs, when incorporated into DNA, produce a covalent DNMT-DNA complex, resulting in inactivated DNMTs by the subsequent degradation of the trapped enzymes, also initiating DNA damage [53]. The data of in vitro and in vivo indicate that 5-azacytidine, and 5’-aza-2’deoxycytidine greatly restored the expression of *RASSF1A*. For instance, the NPC cell line C666-1, which was confirmed to be completely methylated and with no expression of *RASSF1A*, was administrated with 5’-aza-2’deoxycytidine. The restoration of *RASSF1A* was observed in the 5’-aza-2’deoxycytidine-treated cell line [34]. The treatment with 5-azacytidine, as reported, could enhance the radiosensitivity of both the CNE2 and SUNE1 NPC cell lines. Notably, the combination of both 5-azacytidine treatment and irradiation significantly inhibited the growth of the tumor in the mouse xenograft model and enhanced radiation-induced apoptosis in vitro compared to 5-azaC alone or IR alone [54].

Moreover, the determination of *RASSF1A* downstream effectors may provide potential interest for therapeutic regulators in patients with NPC, especially metastatic NPC [55]. According to their study, they reported that the expression of *RASSF1A* inactivates YAP1 by remodeling F-actin assembly, as the results, suppress the transcriptional activity of PDGFB, which was reported as the important element for sustaining the malignant phenotypes of NPC cells. The silence of PDGFB abrogated the RASSF1A depletion-induced malignant phenotypes of NPC cells [55]. Thus, it could be inferred that the inactivation of *RASSF1A*, mechanistically, may, through the epigenetic event of hypermethylation, trigger the process of NPC. Moreover, it is suggested that RASSF1A inhibits malignant phenotypes by repressing PDGFB expression in a YAP1-dependent manner and PDGFB could serve as the potential therapeutic agent for metastatic NPC treatment [55].

## 4. Other Genes Located at 3p Have Also Been Studied though Not as much as *RASSF1A*

Besides *RASSF1A*, other genes, including *Blu*, *RAR-β*, *DLEC1*, and *LTF* have also been studied. A recent report indicated that *Blu* is a stress-responsive gene and is regulated by heat shock and environmental factors. Moreover, the overexpression of *Blu* was reported to inhibit the colony formation of cancer cells [16]. The first evidence of downregulated *Blu* by the aberrant hypermethylation involved in the pathogenesis of NPC was reported by Liu et al. (2003). They found high frequency of promoter hypermethylation in 74% (17 of 23) of primary tumors and 100% of cell lines, including CNE1, CNE2, and HNE1, in contrast to the status in which no methylation was shown in non-cancerous controls. The restoration of *Blu* expression was observed in CNE2 by the treatment of 5’-aza-2’deoxycytidine [19]. The aberrant hypermethylation of *Blu* was reported in Tunisian NPC patients. The frequency of *Blu* was 34.1% and 86.3%, respectively [7]. The frequency of methylation *Blu* in Tunisian NPC patients was lower than in Chinese NPC patients, in which it was 66% (19 of 29 primary tumors) and Vietnamese NPC patients, in which it was 81.11% (73 of 90 NPC tissues) [7,17,20]. It is noted that there was a significant correlation between aberrant methylation of the *Blu* promoter and undifferentiated histological type of NPC [7]. Thus, the aberrant methylation of *Blu* is the common event in the development of NPC.

The aberrant hypermethylation of *DLEC1* was also reported in Tunisian NPC patients with a frequency of 86.3% [7]. The recorded methylation frequency of *DLEC1* in their report was higher than the frequency of 71.43% (30 of 42 NPC primary tumors) in Chinese NPC patients and the frequency of 60.42% (29 of 48 NPC tissues) in NPC patients of Hong Kong [7,39,40]. Only the single-gene DLEC1-based method had a specificity of 96% and sensitivity of 60% in diagnosis of NPC [39]. Sensitivity reached 78% and specificity was unchanged in the case of combining *DLEC1* and *KIF1A* [39]. Similar to previous studies, Tian et al. (2013) also investigated promoter methylation of *DLEC1*, as well as combination with *RASSF1A*, *CDKN2A*, *DAPK*, and *UCHL*1 as the methylation markers in the diagnosis of NPC in serum samples [38]. Thus, screening DNA hypermethylation of *DLEC1* in serum is a promising strategy for the diagnosis of NPC.

*RAR-β* belongs to the nuclear receptor superfamily and it mediates cellular signaling, cell growth, and differentiation. Kwong et al. (2002) reported the aberrant methylation of *RAR-β* in NPC primary tumors, cell lines, and xenograft. Aberrant methylation was significantly observed in 81% (32 of 40 cases) of primary tumor, 75% (3 of 4 cases) of cell lines, and 50% (2 of 4 cases) of xenografts, compared to normal nasopharyngeal epithelium (100% cases without methylation). They also included the finding that the hypermethylated promoter of *RAR-β* may block or interfere with the retinoid signaling pathways in NPC [30]. Later on, a strong association was shown between the expression of *cyclooxygenase* (COX-2) and loss of *RAR-β* via the aberrant methylation in NPC tissues. Their data supported the finding that the inhibition of tumor development of *RAR-β* may be related to its suppression of *COX-2*. The accumulation of COX-2 is related to tumor progression and invasiveness. Additionally, *RAR-β* hypermethylation is correlated with histological type of NPC [36]. Challouf et al. (2012) also reported that aberrant methylation of *RAR-β* was significantly frequent in NPC tumors with lymph node metastasis than those without metastasis [28]. Taken together, these data demonstrate that the loss expression of *RAR-β* by the mechanism of hypermethylation is significantly associated with highly differentiated tumors, advanced tumor stage, and lymph node metastasis. The application of methylation markers of panel genes, including *RASSF1A*, *RAR-β*, *DAPK*, *p16*, *p15*, *p14*, *MGMT*, and *GSTP1*, or *RASSF1A*, *RAR-β*, and *DAPK,* give a specificity of 100% and sensitivity of 100% [30,36]. Thus, it indicates a promising methylation marker for NPC diagnosis.

*LTF* gene behaves as a tumor suppressor gene in NPC by the function of inducing cell rest and modulating the MAPK signaling pathway [14]. Other reports indicate that LTP also represses AKT signaling in NPC through multiple mechanisms [25]. Yi et al. (2006) first identified that the downregulation of *LTF* was observed in 76% (25 of 33 cases) of primary NPC tissues, and the hypermethylation of *LTF* was observed in 63.6% (21 of 33 cases) of primary NPC samples, but not in chronic nasopharyngeal tissues. By treatment of 5’-aza-2’deoxycytidine, the increasing transcript of LTF was found in the NPC cell line. Additionally, they also reported that two-hit silencing of *LTF* through genetic and epigenetic alterations may be a common and key role in nasopharyngeal tumorigenesis [23]. Thus, it may become a biologically relevant diagnosis marker for NPC.

## 5. Conclusions and Future Perspectives

The epigenetic event of hypermethylation plays key roles in nasopharyngeal tumorigenesis. Many studies have been carried out to identify the target TSGs located at 3p which are responsible for NPC development and progress. In this study, we focused on exploiting the available data based on research over the past two decades to identify the potential TGS-located 3p-based methylation markers, particularly *BLU*, *DLEC1*, *LTF*, *RASSF1A*, and *RAR-β*, for NPC diagnosis. These profiles have become a potential component for the use of these TSGs in the design of in vitro assays and evaluation of further studies with a larger cohort of NPC patients to point out the perturbation targets in comparison with healthy volunteers as diagnostic markers in cancer management, including screening activities, monitoring of routine tumorigenesis, and development of TGS-located 3p-based therapies.

## Figures and Tables

**Table 1:** Roles of TSGs on chromosome 3 during nasopharyngeal carcinogenesis.

Gene	Location	Roles	References
*ADAMTS9*	3p14.1	Angiogenesis	[6]
*PTPRG*	3p14.2	Chromosomal translocations and deletions, cell cycle	[18]
*BLU*	3p21.3	Cell progress, stress-response	[7,16,17,19,20]
*GNAT1*	3p21.3	Remains to be revealed	[21]
*LARS2*	3p21.3	Protein synthesis	[22]
*LTF*	3p21.3	Immunomodulatory, homeostasis, anti-tumor activity, cell growth, cell cycle regulatory	[23,24,25]
*MLH1*	3p21.3	Mismatch repair	[26,27]
*RASSF1A*	3p21.3	Cell proliferation, cell cycle regulation, apoptosis, micro-tubular stabilization	[5,13,26,27,28,29,30,31,32,33,34,35,36,37,38]
*ITGA9*	3p21.3	Cell––cell and cell–matrix adhesion	[15]
*DLEC1*	3p22.2	Cell communication, signaling transduction, cell proliferation	[7,38,39,40]
*RAR-β*	3p24.2	Hormone receptor, transcriptional regulator, retinoic acid signaling, cell growth and differentiation	[28,30,36]
*VLH1*	3p25.3	Ubiquitination	[27]
*TIG1*	3q25.3	Cell-to-cell contact	[39,41]

**Table 2:** Specificity, sensitivity, positive predictive value, and negative predictive value of methylated *RASSF1A*-based diagnosis (focused on the case-control study).

NPC Case (n)	Non-Cancerous Control (n)	Case	Control	Sp, Se, Po, Ne	References
Primary tumor tissue (28)	Nasopharyngeal epithelium (6)	14/21 (66.67%)	0/6 (0.00%)	Sp = 100.00% Se = 66.67% Po = 100.00% Ne = 46.15%	[34]
Primary tumor tissue (28)	Tissue (6)	23/28 (82.14%)	0/6 (0.00%)	Sp = 100.00% Se = 82.14% Po = 100.00% Ne = 54.55%	[30]
Nasopharyngeal brushing (28)	Nasopharyngeal brushing (12)	11/28 (39.29%)	0/26 (0.00%)	Sp = 100.00% Se = 39.29% Po = 100.00% Ne = 60.47%	[31]
Primary tumor tissue (30)	Tissue (6)	20/30 (66.67%)	0/6 (0.00%)	Sp = 100.00% Se = 66.67% Po = 100.00% Ne = 37.50%	[29]
Nasopharyngeal brushing (30)	Nasopharyngeal brushing (37)	10/30 (33.33%)	0/37 (0.00%)	Sp = 100.00% Se = 33.33% Po = 100.00% Ne = 64.91%	
M&T rinsing fluid (30)	M&T rinsing fluid (43)	11/30 (36.67%)	0/43 (0.00%)	Sp = 100.00% Se = 36.67% Po = 100.00% Ne = 69.36%	
Plasma (30)	Plasma (43)	9/30 (30.00%)	1/43 (2.32%)	Sp = 97.67% Se = 30.00% Po = 90.00% Ne = 66.67%	
Buffy coat (30)	Buffy coat (43)	0/30 (0.00%)	1/43 (2.32%)	Sp = 97.67% Se = 0.00% Po = 0.00% Ne = 58.33%	
Tumor tissue (28)	Tissue (5)	13/28 (46.43%)	0/5 (0.00%)	Sp = 100.00% Se = 46.43% Po = 100.00% Ne = 25.00%	[27]
Plasma (41)	Plasma (43)	2/41 (4.88%)	0/43 (0.00%)	Sp = 100.00% Se = 4.88% Po = 100.00% Ne = 52.44%	[26]
Tumor tissue (68)	Tissue (9)	62/68 (91.18%)	0/9 (0.00%)	Sp = 100.00% Se = 91.18% Po = 100.00% Ne = 60.00%	[36]
Tumor tissue (38)	Tissue (14)	27/38 (71.05%)	0/14 (0.00%)	Sp = 100.00% Se = 71.05% Po = 100.00% Ne = 56.00%	[33]
Tumor tissue/brushing (53)	Nasopharyngeal brushing (25)	40/53 (75.47%)	1/25 (4.00%)	Sp = 96.00% Se = 45.47% Po = 97.56% Ne = 64.87%	[5]
Tumor tissue (36)	Tissue (19)	27/36 (75.00%)	0/19 (0.00%)	Sp = 100.00% Se = 75.00% Po = 100.00% Ne = 67.86%	[28]
Tumor tissue (49)	Tissue (20)	39/49 (79.59%)	0/20 (0.00%)	Sp = 100.00% Se = 79.59% Po = 100.00% Ne = 66.67%	[32]
Nasopharyngeal brushing (49)	Nasopharyngeal brushing (20)	29/49 (59.18%)	0/20 (0.00%)	Sp = 100.00% Se = 59.18% Po = 100.00% Ne = 50.00%	
Serum (40)	Serum (41)	7/40 (17.50%)	2/41 (4.87%)	Sp = 95.12% Se = 17.50% Po = 77.78% Ne = 54.17%	[38]
Tumor tissue (70)	Nasopharyngeal brushing (70)	47/70 (52.22%)	9/70 (12.86%)	Sp = 87.14% Se = 67.14% Po = 83.93% Ne = 72.62%	[35]

Note: Sp: specificity, Se: sensitivity; Po: positive predictive value; Ne: negative predictive value; M&T rinsing fluid: mouth and throat rinsing fluid.

**Table 3:** *RASSF1A* combined with other useful methylated biomarkers for NPC (focused on the case-control study).

Panel of Genes	NPC Case (n)	Non-Cancerous Control (n)	MI	Sp, Se, Po, Ne	References
*RASSF1A*, *RAR-β*, *DAPK*, *p16*, *p15*, *p14*, *MGMT*, *GSTP1*	Primary tumor tissue (28)	Nasopharyngeal epithelium (6)	28/28 (100.00%) of cases, at least one of seven genes: *RASSF1A*, *RAR-β*, *DAPK*, *p16*, *p15*, *p14*, and *MGMT*. All controls were not unmethylated.	Sp = 100.00% Se = 100.00% Po = 100.00% Ne = 100.00%	[30]
*RASSF1A*, *DAPK*, *p16*	Nasopharyngeal brushing (28)	Nasopharyngeal brushing (12)	22/28 (78.57%) of cases, at least one of three genes. All controls were not unmethylated.	Sp = 78.57% Se = 100.00% Po = 100.00% Ne = 66.67%	[31]
*RASSF1A*, *E-cadherin*, *DAPK*, *p15*, *p16*	Primary tumor tissue (30)	Tissue (6)	29/30 of cases, at least one of three genes. All controls were not unmethylated.	Sp = 100.00% Se = 96.97% Po = 100.00% Ne = 85.71%	[29]
	Nasopharyngeal brushing (30)	Nasopharyngeal brushing (37)	24/30 (80.00%) of cases, at least one of three genes. All controls were not unmethylated.	Sp = 100.00% Se = 80.00% Po = 100.00% Ne = 86.05%	
	M&T rinsing fluid (30)	M&T rinsing fluid (43)	26/30 (87.00%) of cases, at least one of three genes. 1/43 (2.32%) of controls, at least one of three genes.	Sp = 97.67% Se = 86.67% Po = 96.30% Ne = 91.30%	
	Plasma (30)	Plasma (43)	3/30 (10.00%) of cases, at least one of three genes. 2/43 (4.65%) of controls, at least one of three genes.	Sp = 95.34% Se = 10.00% Po = 60.00% Ne = 60.30%	
	Buffy coat (30)	Buffy coat (43)	12/30 (40.00%) of cases, at least one of three genes. 3/43 (6.97%) of controls, at least one of three genes.	Sp = 93.02% Se = 40.00% Po = 80.00% Ne = 68.97%	
*RASSF1A*, *MLH1*, *CDH1*, *CDK2B*, *THBS1*, *MGMT*, *CDKN2A*, *TP73*, *C8*, *ARF*, *VHL*	Tumor tissue (28)	Tissue (5)	26/28 (92.86%) of cases, at least one of ten genes: *RASSF1A*, *MLH1*, *CDH1*, *CDK2B*, *THBS1*, *MGMT*, *CDKN2A*, *TP73*, *C8*, and *ARF*. All controls were not unmethylated.	Sp = 100.00% Se = 92.86% Po = 100.00% Ne = 71.43%	[27]
*RASSF1A*, *MLH1*, *CDH1*, *DAPK*, *p15*, *p16*	Plasma (41)	Plasma (43)	29/41 (70.73%) of cases, at least one of six genes. 4/43 (9.30%) of controls, at least one of six genes.	Sp = 90.70% Se = 70.73% Po = 87.88% Ne = 76.47%	[26]
*RASSF1A*, *RAR-β*, *DAPK*	Tumor tissue (68)	Tissue (9)	67/68 (98.53%) of cases, at least one of three genes. All controls were not unmethylated.	Sp = 100.00% Se = 91.18% Po = 100.00% Ne = 60.00%	[36]
*RASSF1A*, *CHFR*, *RIZ1*, *WIFI1*, *p16*, *RASSF2A*, *DAPK1*, *DLC1*, *CDH13*, *CADM1*	Tumor tissue/brushing (53)	Nasopharyngeal brushing (25)	52/53 (98.11%) of cases, at least one of ten genes. nc in controls.	nc	[5]
*RASSF1A*, *RAR-β, SHP1*, *DAPK*, *p16*, *GSTP1*, *TIMP3*, *APC*, *CDH1*, *MGMT*	Tumor tissue (36)	Tissue (19)	nc	nc	[28]
*RASSF1A*, *DAPK*	Tumor tissue (49)	Tissue (20)	nc	nc	[32]
	Nasopharyngeal brushing (49)	Nasopharyngeal brushing (20)	nc	nc	
*RASSF1A*, *DLEC1*, *CDKN2A*, *DAPK*, *UCHL*1	Serum (40)	Serum (41)	34/40 (85.00%) of cases, at least one of five genes. 15/41 (36.59%) of controls, at least one of five genes.	Sp = 85.00% Se = 63.42% Po = 69.39% Ne = 81.25%	[38]

Note: MI: methylation index; Sp: specificity; Se: sensitivity; Po: positive predictive value; Ne: negative predictive value; M&T rinsing fluid: mouth and throat rinsing fluid, nc: no record/non-calculation. Genes in bold were located on 3p.

## Data Availability

Not applicable.
